# Assessing Associations between the *AURKA-HMMR-TPX2-TUBG1* Functional Module and Breast Cancer Risk in *BRCA1/2* Mutation Carriers

**DOI:** 10.1371/journal.pone.0120020

**Published:** 2015-04-01

**Authors:** Ignacio Blanco, Karoline Kuchenbaecker, Daniel Cuadras, Xianshu Wang, Daniel Barrowdale, Gorka Ruiz de Garibay, Pablo Librado, Alejandro Sánchez-Gracia, Julio Rozas, Núria Bonifaci, Lesley McGuffog, Vernon S. Pankratz, Abul Islam, Francesca Mateo, Antoni Berenguer, Anna Petit, Isabel Català, Joan Brunet, Lidia Feliubadaló, Eva Tornero, Javier Benítez, Ana Osorio, Teresa Ramón y Cajal, Heli Nevanlinna, Kristiina Aittomäki, Banu K. Arun, Amanda E. Toland, Beth Y. Karlan, Christine Walsh, Jenny Lester, Mark H. Greene, Phuong L. Mai, Robert L. Nussbaum, Irene L. Andrulis, Susan M. Domchek, Katherine L. Nathanson, Timothy R. Rebbeck, Rosa B. Barkardottir, Anna Jakubowska, Jan Lubinski, Katarzyna Durda, Katarzyna Jaworska-Bieniek, Kathleen Claes, Tom Van Maerken, Orland Díez, Thomas V. Hansen, Lars Jønson, Anne-Marie Gerdes, Bent Ejlertsen, Miguel de la Hoya, Trinidad Caldés, Alison M. Dunning, Clare Oliver, Elena Fineberg, Margaret Cook, Susan Peock, Emma McCann, Alex Murray, Chris Jacobs, Gabriella Pichert, Fiona Lalloo, Carol Chu, Huw Dorkins, Joan Paterson, Kai-Ren Ong, Manuel R. Teixeira, Teixeira, Frans B. L. Hogervorst, Annemarie H. van der Hout, Caroline Seynaeve, Rob B. van der Luijt, Marjolijn J. L. Ligtenberg, Peter Devilee, Juul T. Wijnen, Matti A. Rookus, Hanne E. J. Meijers-Heijboer, Marinus J. Blok, Ans M. W. van den Ouweland, Cora M. Aalfs, Gustavo C. Rodriguez, Kelly-Anne A. Phillips, Marion Piedmonte, Stacy R. Nerenstone, Victoria L. Bae-Jump, David M. O'Malley, Elena S. Ratner, Rita K. Schmutzler, Barbara Wappenschmidt, Kerstin Rhiem, Christoph Engel, Alfons Meindl, Nina Ditsch, Norbert Arnold, Hansjoerg J. Plendl, Dieter Niederacher, Christian Sutter, Shan Wang-Gohrke, Doris Steinemann, Sabine Preisler-Adams, Karin Kast, Raymonda Varon-Mateeva, Andrea Gehrig, Anders Bojesen, Inge Sokilde Pedersen, Lone Sunde, Uffe Birk Jensen, Mads Thomassen, Torben A. Kruse, Lenka Foretova, Paolo Peterlongo, Loris Bernard, Bernard Peissel, Giulietta Scuvera, Siranoush Manoukian, Paolo Radice, Laura Ottini, Marco Montagna, Simona Agata, Christine Maugard, Jacques Simard, Penny Soucy, Andreas Berger, Anneliese Fink-Retter, Christian F. Singer, Christine Rappaport, Daphne Geschwantler-Kaulich, Muy-Kheng Tea, Georg Pfeiler, BCFR, Esther M. John, Alex Miron, Susan L. Neuhausen, Mary Beth Terry, Wendy K. Chung, Mary B. Daly, David E. Goldgar, Ramunas Janavicius, Cecilia M. Dorfling, Elisabeth J. van Rensburg, Florentia Fostira, Irene Konstantopoulou, Judy Garber, Andrew K. Godwin, Edith Olah, Steven A. Narod, Gad Rennert, Shani Shimon Paluch, Yael Laitman, Eitan Friedman, SWE-BRCA, Annelie Liljegren, Johanna Rantala, Marie Stenmark-Askmalm, Niklas Loman, Evgeny N. Imyanitov, Ute Hamann, kConFab Investigators, Amanda B. Spurdle, Sue Healey, Jeffrey N. Weitzel, Josef Herzog, David Margileth, Chiara Gorrini, Manel Esteller, Antonio Gómez, Sergi Sayols, Enrique Vidal, Holger Heyn, GEMO, Dominique Stoppa-Lyonnet, Melanie Léoné, Laure Barjhoux, Marion Fassy-Colcombet, Antoine de Pauw, Christine Lasset, Sandra Fert Ferrer, Laurent Castera, Pascaline Berthet, François Cornelis, Yves-Jean Bignon, Francesca Damiola, Sylvie Mazoyer, Olga M. Sinilnikova, Christopher A. Maxwell, Joseph Vijai, Mark Robson, Noah Kauff, Marina J. Corines, Danylko Villano, Julie Cunningham, Adam Lee, Noralane Lindor, Conxi Lázaro, Douglas F. Easton, Kenneth Offit, Georgia Chenevix-Trench, Fergus J. Couch, Antonis C. Antoniou, Miguel Angel Pujana

**Affiliations:** 1 Hereditary Cancer Program, Catalan Institute of Oncology (ICO), Bellvitge Institute for Biomedical Research (IDIBELL), L’Hospitalet del Llobregat, Catalonia, Spain; 2 Epidemiological Study of Familial Breast Cancer (EMBRACE), Centre for Cancer Genetic Epidemiology, Department of Public Health and Primary Care, University of Cambridge, Strangeways Research Laboratory, Cambridge, United Kingdom; 3 Statistics Unit, Bellvitge Institute for Biomedical Research (IDIBELL), L’Hospitalet del Llobregat, Catalonia, Spain; 4 Department of Laboratory Medicine and Pathology, Mayo Clinic, Rochester, United States of America; 5 Breast Cancer and Systems Biology Unit, Catalan Institute of Oncology (ICO), Bellvitge Institute for Biomedical Research (IDIBELL), L’Hospitalet del Llobregat, Catalonia, Spain; 6 Department of Genetics and Biodiversity Research Institute (IRBio), University of Barcelona, Barcelona, Catalonia, Spain; 7 Department of Health Sciences Research, Mayo Clinic, Rochester, Minnesota, United States of America; 8 Department of Genetic Engineering and Biotechnology, University of Dhaka, Dhaka, Bangladesh; 9 Department of Pathology, University Hospital of Bellvitge, Bellvitge Institute for Biomedical Research (IDIBELL), L’Hospitalet del Llobregat, Catalonia, Spain; 10 Hereditary Cancer Program, Catalan Institute of Oncology (ICO), Girona Biomedical Research Institute (IDIBGI), Hospital Josep Trueta, Girona, Catalonia, Spain; 11 Human Genetics Group, Spanish National Cancer Centre (CNIO), and Biomedical Network on Rare Diseases, Madrid, Spain; 12 Oncology Service, Hospital de la Santa Creu i Sant Pau, Barcelona, Catalonia, Spain; 13 Department of Obstetrics and Gynecology, University of Helsinki and Helsinki University Central Hospital, Helsinki, Finland; 14 Department of Clinical Genetics, Helsinki University Central Hospital, Helsinki, Finland; 15 Division of Cancer Medicine, University of Texas MD Anderson Cancer Center, Houston, Texas, United States of America; 16 Division of Human Cancer Genetics, Departments of Internal Medicine and Molecular Virology, Immunology and Medical Genetics, Comprehensive Cancer Center, The Ohio State University, Columbus, Ohio, United States of America; 17 Women's Cancer Program at the Samuel Oschin Comprehensive Cancer Institute, Cedars-Sinai Medical Center, Los Angeles, California, United States of America; 18 Clinical Genetics Branch, Division of Cancer Epidemiology and Genetics, National Cancer Institute, Maryland, Rockville, United States of America; 19 Department of Medicine and Genetics, University of California San Francisco, San Francisco, California, United States of America; 20 Samuel Lunenfeld Research Institute, Mount Sinai Hospital, and Departments of Molecular Genetics and Laboratory Medicine and Pathobiology, University of Toronto, Toronto, Ontario, Canada; 21 Abramson Cancer Center and Department of Medicine, The University of Pennsylvania School of Medicine, Philadelphia, Pennsylvania, United States of America; 22 Abramson Cancer Center and Center for Clinical Epidemiology and Biostatistics, The University of Pennsylvania Perelman School of Medicine, Philadelphia, Pennsylvania, United States of America; 23 Department of Pathology, Landspitali University Hospital and BMC, Faculty of Medicine, University of Iceland, Reykjavik, Iceland; 24 Department of Genetics and Pathology, Pomeranian Medical University, Szczecin, Poland; 25 Center for Medical Genetics, Ghent University, Ghent, Belgium; 26 Oncogenetics Group, Vall d’Hebron Institute of Oncology (VHIO), Vall d’Hebron Research Institute (VHIR) and Universitat Autònoma de Barcelona, Barcelona, Catalonia, Spain; 27 Center for Genomic Medicine, Rigshospitalet, Copenhagen University Hospital, Copenhagen, Denmark; 28 Department of Clinical Genetics, Rigshospitalet, Copenhagen University Hospital, Copenhagen, Denmark; 29 Department of Oncology, Rigshospitalet, Copenhagen University Hospital, Copenhagen, Denmark; 30 Molecular Oncology Laboratory, Hospital Clínico San Carlos, San Carlos Research Institute (IdISSC), Madrid, Spain; 31 All Wales Medical Genetics Service, Glan Clwyd Hospital, Rhyl, United Kingdom; 32 All Wales Medical Genetics Services, Singleton Hospital, Swansea, United Kingdom; 33 Clinical Genetics, Guy’s and St. Thomas’ National Health Service (NHS) Foundation Trust, London, United Kingdom; 34 Genetic Medicine, Manchester Academic Health Sciences Centre, Central Manchester University Hospitals National Health Service (NHS) Foundation Trust, Manchester, United Kingdom; 35 Yorkshire Regional Genetics Service, Leeds, United Kingdom; 36 North West Thames Regional Genetics Service, Kennedy-Galton Centre, Harrow, United Kingdom; 37 Department of Clinical Genetics, East Anglian Regional Genetics Service, Addenbrookes Hospital, Cambridge, United Kingdom; 38 West Midlands Regional Genetics Service, Birmingham Women’s Hospital Healthcare National Health Service (NHS) Trust, Edgbaston, Birmingham, United Kingdom; 39 Department of Genetics, Portuguese Oncology Institute, and Biomedical Sciences Institute (ICBAS), Porto University, Porto, Portugal; 40 Hereditary Breast and Ovarian Cancer Research Group Netherlands (HEBON), Netherlands Cancer Institute (NKI), Amsterdam, The Netherlands; 41 Family Cancer Clinic, Netherlands Cancer Institute (NKI), Amsterdam, The Netherlands; 42 Department of Genetics, University Medical Centre Groningen, University of Groningen, Groningen, Netherlands; 43 Department of Medical Oncology, Family Cancer Clinic, Erasmus University Medical Center, Rotterdam, The Netherlands; 44 Department of Medical Genetics, University Medical Center Utrecht, Utrecht, The Netherlands; 45 Department of Human Genetics and Department of Pathology, Radboud university medical center, Nijmegen, The Netherlands; 46 Department of Human Genetics, Leiden University Medical Center, Leiden, The Netherlands; 47 Department of Pathology, Leiden University Medical Center, Leiden, The Netherlands; 48 Department of Clinical Genetics, Leiden University Medical Center, Leiden, The Netherlands; 49 Department of Epidemiology, Netherlands Cancer Institute, Amsterdam, The Netherlands; 50 Department of Clinical Genetics, Vrije Universiteit (VU) University Medical Centre, Amsterdam, The Netherlands; 51 Department of Clinical Genetics, Maastricht University Medical Center, Maastricht, The Netherlands; 52 Department of Clinical Genetics, Erasmus University Medical Center, Rotterdam, The Netherlands; 53 Department of Clinical Genetics, Academic Medical Center, Amsterdam, The Netherlands; 54 Division of Gynecologic Oncology, NorthShore University HealthSystem, University of Chicago, Chicago, Illinois, United States of America; 55 Division of Cancer Medicine, Peter MacCallum Cancer Centre, East Melbourne, Victoria, Australia; 56 Gynecologic Oncology Group Statistical and Data Center, Roswell Park Cancer Institute, Buffalo, New York, United States of America; 57 Central Connecticut Cancer Consortium, Hartford Hospital/Helen and Harry Gray Cancer Center, Hartford, Connecticut, United States of America; 58 Division of Gynecologic Oncology, Department of Obstetrics and Gynecology, University of North Carolina, Chapel Hill, North Carolina, United States of America; 59 Division of Gynecologic Oncology, Ohio State University, Columbus Cancer Council, Hilliard, Ohio, United States of America; 60 Division of Gynecologic Oncology, Yale University School of Medicine, New Haven, Connecticut, United States of America; 61 Centre of Familial Breast and Ovarian Cancer and Centre for Integrated Oncology (CIO), University Hospital of Cologne, Cologne, Germany; 62 Institute for Medical Informatics, Statistics and Epidemiology, University of Leipzig, Leipzig, Germany; 63 Department of Gynecology and Obstetrics, Division of Tumor Genetics, Klinikum Rechts der Isar, Technical University Munich, Munich, Germany; 64 Department of Gynecology and Obstetrics, Ludwig-Maximilian University Munich, Munich, Germany; 65 Department of Gynecology and Obstetrics, Christian-Albrechts-University of Kiel University Medical Center Schleswig-Holstein, Kiel, Germany; 66 Institute of Human Genetics, Christian-Albrechts-University of Kiel University Medical Center Schleswig-Holstein, Kiel, Germany; 67 Department of Gynecology and Obstetrics, University Hospital Düsseldorf, Heinrich-Heine University Düsseldorf, Düsseldorf, Germany; 68 Institute of Human Genetics, Department of Human Genetics, University Hospital Heidelberg, Heidelberg, Germany; 69 Department of Gynecology and Obstetrics, University Hospital Ulm, Ulm, Germany; 70 Institute of Cell and Molecular Pathology, Hannover Medical School, Hannover, Germany; 71 Institute of Human Genetics, University of Münster, Münster, Germany; 72 Department of Gynecology and Obstetrics, University Hospital Carl Gustav Carus, Technical University Dresden, Dresden, Germany; 73 Institute of Human Genetics, Campus Virchov Klinikum, Charite Berlin, Berlin, Germany; 74 Centre of Familial Breast and Ovarian Cancer, Department of Medical Genetics, Institute of Human Genetics, University Würzburg, Würzburg, Germany; 75 Department of Clinical Genetics, Vejle Hospital, Vejle, Denmark; 76 Section of Molecular Diagnostics, Department of Biochemistry, Aalborg University Hospital, Aalborg, Denmark; 77 Department of Clinical Genetics, Aarhus University Hospital, Aarhus, Denmark; 78 Department of Clinical Genetics, Odense University Hospital, Odense, Denmark; 79 Department of Cancer Epidemiology and Genetics, Masaryk Memorial Cancer Institute, Brno, Czech Republic; 80 Fondazione Istituto di Oncologia Molecolare (IFOM), Fondazione Italiana per la Ricerca sul Cancro (FIRC), Milan, Italy; 81 Department of Experimental Oncology, Istituto Europeo di Oncologia (IEO), Cogentech Cancer Genetic Test Laboratory, Milan, Italy; 82 Unit of Medical Genetics, Department of Preventive and Predictive Medicine, Istituto di Ricovero e Cura a Carattere Scientifico (IRCCS), Fondazione Istituto Nazionale Tumori (INT), Milan, Italy; 83 Unit of Molecular Bases of Genetic Risk and Genetic Testing, Department of Preventive and Predictive Medicine, Istituto di Ricovero e Cura a Carattere Scientifico (IRCCS), Fondazione Istituto Nazionale Tumori (INT), Milan, Italy; 84 Department of Molecular Medicine, "Sapienza" University, Rome, Italy; 85 Immunology and Molecular Oncology Unit, Istituto Oncologico Veneto (IOV), Istituto di Ricovero e Cura a Carattere Scientifico (IRCCS), Padua, Italy; 86 Laboratoire de Diagnostic Génétique et Service d'Onco-Hématologie, Hopitaux Universitaire de Strasbourg, Centre Hospitalier Régional Universitaire (CHRU) Nouvel Hôpital Civil, Strasbourg, France; 87 Cancer Genomics Laboratory, Centre Hospitalier Universitaire de Québec Research Center and Laval University, Quebec City, Canada; 88 Department of Gynecology and Obstetrics, and Comprehensive Cancer Center, Medical University of Vienna, Vienna, Austria; 89 Breast Cancer Family Registry (BCFR), Cancer Prevention Institute of California, Fremont, California, United States of America; 90 Department of Epidemiology, Cancer Prevention Institute of California, Fremont, California, United States of America; 91 Department of Cancer Biology, Dana-Farber Cancer Institute, Boston, Massachusetts, United States of America; 92 Department of Population Sciences, Beckman Research Institute of City of Hope, Duarte, California, United States of America; 93 Department of Epidemiology, Columbia University, New York, New York, United States of America; 94 Departments of Pediatrics and Medicine, Columbia University Medical Center, New York, New York, United States of America; 95 Department of Clinical Genetics, Fox Chase Cancer Center, Philadelphia, Pennsylvania, United States of America; 96 Department of Dermatology, University of Utah School of Medicine, Salt Lake City, Utah, United States of America; 97 Vilnius University Hospital Santariskiu Clinics, Hematology, Oncology and Transfusion Medicine Center, Department of Molecular and Regenerative Medicine, State Research Centre Institute for Innovative medicine, Vilnius, Lithuania; 98 Cancer Genetics Laboratory, Department of Genetics, University of Pretoria, Arcadia, South Africa; 99 Molecular Diagnostics Laboratory, Institute of Radioisotopes and Radiodiagnostic Products (IRRP), National Centre for Scientific Research Demokritos, Athens, Greece; 100 Center for Cancer Genetics and Prevention, Dana-Farber Cancer Institute, Harvard Medical School, Boston, Massachusetts, United States of America; 101 Department of Pathology and Laboratory Medicine, University of Kansas Medical Center, Kansas City, Kansas, United States of America; 102 Department of Molecular Genetics, National Institute of Oncology, Budapest, Hungary; 103 Women's College Research Institute, University of Toronto, Toronto, Canada; 104 Clalit National Israeli Cancer Control Center and Department of Community Medicine and Epidemiology, Carmel Medical Center and B Rappaport Faculty of Medicine, Haifa, Israel; 105 The Institute of Oncology, Chaim Sheba Medical Center, Ramat Gan, Israel; 106 The Susanne Levy Gertner Oncogenetics Unit, Institute of Human Genetics, Chaim Sheba Medical Center, Ramat Gan, Israel; 107 Sackler Faculty of Medicine, Tel Aviv University, Ramat Aviv, Israel; 108 Swedish BRCA1 and BRCA2 Study (SWE-BRCA), Stockholm, Sweden; 109 Department of Oncology, Karolinska University Hospital, Stockholm, Sweden; 110 Department of Clinical Genetics, Karolinska University Hospital, Stockholm, Sweden; 111 Division of Clinical Genetics, Department of Clinical and Experimental Medicine, Linköping University, Linköping, Sweden; 112 Department of Oncology, Lund University Hospital, Lund, Sweden; 113 N.N. Petrov Institute of Oncology, St.-Petersburg, Russia; 114 Molecular Genetics of Breast Cancer, German Cancer Research Center (DKFZ), Heidelberg, Germany; 115 Kathleen Cuningham Consortium for Research into Familial Breast Cancer (kConFab), Peter MacCallum Cancer Center, Melbourne, Australia; 116 Queensland Institute of Medical Research (QIMR) Berghofer Medical Research Institute, Brisbane, Australia; 117 Clinical Cancer Genetics, City of Hope, Duarte, California, United States of America; 118 St. Joseph Hospital of Orange, Care of City of Hope Clinical Cancer Genetics Community Research Network, Duarte, California, United States of America; 119 The Campbell Family Institute for Breast Cancer Research, Ontario Cancer Institute, University Health Network, Toronto, Canada; 120 Cancer Epigenetics and Biology Program (PEBC), IDIBELL, L’Hospitalet del Llobregat, Catalonia, Spain; 121 Department of Physiological Sciences II, School of Medicine, University of Barcelona, L’Hospitalet del Llobregat, Catalonia, Spain; 122 Catalan Institution for Research and Advanced Studies (ICREA), Barcelona, Catalonia, Spain; 123 Groupe Genetique et Cancer (GEMO), National Cancer Genetics Network, French Federation of Comprehensive Cancer Centers (UNICANCER), Paris, France; 124 Department of Tumour Biology, Institut Curie, Paris, France; 125 Institut National de la Santé et de la Recherche Médicale (INSERM) U830, Institut Curie, Paris, France; 126 Université Paris Descartes, Sorbonne Paris Cité, Paris, France; 127 Unité Mixte de Génétique Constitutionnelle des Cancers Fréquents, Hospices Civils de Lyon–Centre Léon Bérard, Lyon, France; 128 Institut National de la Santé et de la Recherche Médicale (INSERM) U1052, Centre National de la Recherche Scientifique (CNRS) UMR5286, Université Lyon 1, Centre de Recherche en Cancérologie de Lyon, Lyon, France; 129 Université Lyon 1, Centre National de la Recherche Scientifique (CNRS) UMR5558, and Unité de Prévention et d’Epidémiologie Génétique, Centre Léon Bérard, Lyon, France; 130 Laboratoire de Génétique Chromosomique, Hôtel Dieu Centre Hospitalier, Chambéry, France; 131 Centre François Baclesse, Caen, France; 132 Genetic Unit, Avicenne Hospital, Assitance Publique-Hôpitaux de Paris, Paris, Sud-Francilien Hospital, Evry-Corbeil, and University Hospital, Clermont-Ferrand, France; 133 Département d'Oncogénétique, Centre Jean Perrin, Université de Clermont-Ferrand, Clermont-Ferrand, France; 134 Department of Pediatrics, Child and Family Research Institute, University of British Columbia, Vancouver, Canada; 135 Clinical Genetics Research Laboratory, Memorial Sloan-Kettering Cancer Center, New York, New York, United States of America; 136 Department of Oncology, Mayo Clinic, Rochester, Minnesota, United States of America; 137 Center for Individualized Medicine, Mayo Clinic, Scottsdale, Arizona, United States of America; Sapporo Medical University, JAPAN

## Abstract

While interplay between BRCA1 and AURKA-RHAMM-TPX2-TUBG1 regulates mammary epithelial polarization, common genetic variation in *HMMR* (gene product RHAMM) may be associated with risk of breast cancer in *BRCA1* mutation carriers. Following on these observations, we further assessed the link between the *AURKA-HMMR-TPX2-TUBG1* functional module and risk of breast cancer in *BRCA1* or *BRCA2* mutation carriers. Forty-one single nucleotide polymorphisms (SNPs) were genotyped in 15,252 *BRCA1* and 8,211 *BRCA2* mutation carriers and subsequently analyzed using a retrospective likelihood approach. The association of *HMMR* rs299290 with breast cancer risk in BRCA1 mutation carriers was confirmed: per-allele hazard ratio (HR) = 1.10, 95% confidence interval (CI) 1.04 – 1.15, p = 1.9 x 10^−4^ (false discovery rate (FDR)-adjusted p = 0.043). Variation in *CSTF1*, located next to *AURKA*, was also found to be associated with breast cancer risk in *BRCA2* mutation carriers: rs2426618 per-allele HR = 1.10, 95% CI 1.03 – 1.16, p = 0.005 (FDR-adjusted p = 0.045). Assessment of pairwise interactions provided suggestions (FDR-adjusted p_interaction_ values > 0.05) for deviations from the multiplicative model for rs299290 and *CSTF1* rs6064391, and rs299290 and *TUBG1* rs11649877 in both *BRCA1* and *BRCA2* mutation carriers. Following these suggestions, the expression of *HMMR* and *AURKA* or *TUBG1* in sporadic breast tumors was found to potentially interact, influencing patients’ survival. Together, the results of this study support the hypothesis of a causative link between altered function of AURKA-HMMR-TPX2-TUBG1 and breast carcinogenesis in *BRCA1/2* mutation carriers.

## Introduction

An integrative genomics study generated a breast cancer network model that predicted novel genetic and molecular relationships for breast cancer tumor suppressors [1]. Among the predictions, the product of the hyaluronan-mediated motility receptor (*HMMR*) gene, RHAMM, was found to be biochemically and functionally linked to the breast cancer gene, early onset 1 gene product (BRCA1) [1]. Analysis of common genetic variation in *HMMR* suggested an association with breast cancer risk in Ashkenazi Jewish women, with a greater increased risk in younger individuals [1]. However, this association was not observed either in a European case-control study [2] or in a genome-wide association study in postmenopausal women of European ancestry [3].

Following the initial functional evidence, a molecular mechanism involving RHAMM and BRCA1 was found to regulate mammary epithelial apicobasal polarization and, possibly, differentiation [4]. The results from this study indicated that RHAMM and BRCA1 play a central role in the cytoskeletal reorganization necessary for epithelial polarization. This functional interplay included interactions with the product of the proto-oncogene aurora kinase A (AURKA) and its major regulator, targeting protein for Xklp2 (TPX2), in addition to g-tubulin (TUBG1) [4]. In this scenario, cell proliferation is endorsed by activated AURKA while polarization and differentiation are mediated by activation of BRCA1 and degradation of RHAMM. Intriguingly, the same *HMMR* variation as originally detected in the Ashkenazi Jewish population was suggested to be associated with breast cancer risk in *BRCA1*, but not in *BRCA2* mutation carriers [4]. This observation was endorsed by complementary analyses in breast cancer tissue; specifically, loss of cell polarity was revealed in *in situ* breast tissue lesions of *BRCA1* mutation carriers and, accordingly, increased staining of phospho-T703-RHAMM (target of AURKA) was preferentially detected in estrogen receptor α (ERα)-negative and *BRCA1*-mutated tumors [4].

While the *HMMR* association study in *BRCA1/2* mutation carriers drew on a partial dataset from the Consortium of Investigators of Modifiers of *BRCA1/2* (CIMBA), the depicted mechanistic model highlighted additional gene candidates for breast cancer risk; i.e., *AURKA*, *TPX2*, and *TUBG1* [4]. In a previous CIMBA study, no evidence of association was found between functional variation in *AURKA* and breast cancer risk among *BRCA1/2* mutation carriers [5]. However, these results were based on a more limited CIMBA dataset (4,935 *BRCA1* and 2,241 *BRCA2* mutation carriers) and did not comprehensively assess variation in the *AURKA* genomic region. Furthermore, variation in *TUBG1* was found to be associated with breast cancer risk in a hospital-based case-control study [6], but has not been assessed in *BRCA1/2* mutation carriers.

In addition to multiplicative allele effects, systematic analyses in model organisms have shown that a given phenotype may be substantially determined by genetic interactions (GxG); that is, “epistasis” in statistical terms, defined as deviation from additivity for a quantitative phenotype arising from the effect of genetic variants or mutations in another locus [7]. Importantly, GxG significantly overlap with other types of gene and/or protein relationships [8–10]. Therefore, the functional interplay between the aforementioned genes/proteins in a key mammary epithelial cell process could support the existence of genetic interactions that influence cancer risk.

In the present study, given previous evidence of 1) the functional interplay between BRCA1 and AURKA-RHAMM-TPX2-TUBG1 in mammary epithelial polarization [4], and 2) the potential modification of breast cancer risk in *BRCA1* mutation carriers by common genetic variation in *HMMR* [4], we further assessed the association between variants in *AURKA-HMMR-TPX2-TUBG1* and breast cancer risk in *BRCA1* and *BRCA2* mutation carriers. Genotyped variants from the custom Illumina iSelect array of the Collaborative Oncological Gene-environment Study (iCOGS) were analyzed in a large series of *BRCA1/2* mutation carriers [11,12].

## Materials and Methods

### Study Subjects and Ethics Statement

*BRCA1/2* mutation carriers were recruited under the CIMBA initiative following approval of the corresponding protocol by the institutional review board or ethics committee at each participating center, and written informed consent was obtained from the patients when required [11,12]. Sixty CIMBA study centers recruited 15,252 *BRCA1* and 8,211 *BRCA2* mutation carriers that passed quality control assessment in this study. Most of these individuals were recruited through cancer genetics clinics and enrolled into national or regional studies. The remaining carriers were identified by population-based sampling or community recruitment. Eligibility in CIMBA was restricted to female carriers of pathogenic *BRCA1* and *BRCA2* mutations who were ≥ 18 years old at recruitment. Information collected included year of birth, mutation description, self-reported ethnicity, age at last follow-up, ages at breast or ovarian cancer diagnosis, and age at bilateral prophylactic mastectomy or oophorectomy. Information regarding tumor characteristics, including ERα status, was collected for 3,458 *BRCA1* and 1,924 *BRCA2* mutation carriers. Related individuals were identified by a unique family identifier.

### iCOGS Design

The iCOGS array, genotyping and quality controls for the CIMBA *BRCA1/2* mutation carrier samples have been described recently [11,12]. The final array design included 211,155 manufactured SNPs that were selected on the basis of primary evidence from genome-wide association studies (GWASs) of breast, ovarian and prostate cancer, for fine mapping of known cancer susceptibility loci, and included functional candidate variants of interest [11–15] (also see http://www.nature.com/icogs/primer/cogs-project-and-design-of-the-icogs-array/ and http://ccge.medschl.cam.ac.uk/research/consortia/icogs/). Details of the iCOGS array design have been described elsewhere [11–15]. The genotype data used in this study are available upon request from the CIMBA Data Access Coordinating Committee (contact A.C.A.).

### Association Study

Based on previous GWAS results for *BRCA1* [16] and *BRCA2* [17] mutation carriers, and on the selection of gene candidates, the iCOGS array included SNPs in the *AURKA* (n = 15), *HMMR* (n = 14), *TPX2* (n = 3) and *TUBG1* (n = 4) loci (defined as ± 20 kilobases (kb) from the genomic structure of each gene), and these were analyzed in the present study (**S1 Table**). In addition, we analyzed five SNPs proximal to the *HMMR* locus that provided some suggestion of association with breast cancer risk in Ashkenazi Jewish women [1] (**S1 Table**). In total, these SNPs represented 32 partially independent variants (pairwise r^2^ < 0.85). To account for multiple testing, we used a FDR approach for the 41 genotyped SNPs that were evaluated for their associations with breast cancer risk in *BRCA1* and *BRCA2* mutation carriers; significant results are reported for FDR < 5%. The main analyses focused on evaluating associations between each genotype and breast cancer or ovarian cancer risk separately, in a survival analysis framework. In the breast cancer analysis, the phenotype of each individual was defined by age at breast cancer diagnosis or age at last follow-up. Individuals were followed until the age of the first breast or ovarian cancer diagnosis or bilateral prophylactic mastectomy, whichever occurred first, or until age at last observation. Mutation carriers censored at ovarian cancer diagnosis were considered to be unaffected. For the ovarian cancer analysis, the primary endpoint was the age at ovarian cancer diagnosis, and mutation carriers were followed until the age of ovarian cancer diagnosis or risk-reducing salpingo-oophorectomy, or until age at last observation. In order to maximize the number of ovarian cancer cases, breast cancer was not considered to be a censoring event in this analysis, and mutation carriers who developed ovarian cancer after breast cancer diagnosis were considered affected in the ovarian cancer analysis. To adjust for the non-random sampling of mutation carriers with respect to their disease status, data were analyzed by modeling the retrospective likelihood of the observed genotypes conditional on the disease phenotypes [18]. The associations were assessed using the 1-degree of freedom score test statistic based on this retrospective likelihood. To allow for the non-independence among related individuals, the correlation between the genotypes was taken into account using a kinship-adjusted version of the score test statistic [16]. The p values presented were based on the adjusted score test. To estimate the HRs, the effect of each SNP was modeled as either a per-allele or genotype on the log-scale by maximizing the retrospective likelihood. The evidence of heterogeneity in the associations between countries/study-centers was also evaluated. Associations with breast and ovarian cancer risks were assessed simultaneously within a competing risk analysis framework [11,18]. The significant FDR-adjusted associations (for rs299290 in *HMMR* and for rs2426618 in *AURKA*/*CSTF1*) were subsequently explored using imputed genotypes based on data from the 1,000 Genomes project (March 2012 version [19]). The IMPUTE2 software [20] was used for imputation of non-genotyped SNPs. Associations of each marker with cancer risk were assessed using a similar score test to that used for the genotyped SNPs, but based on the posterior genotype probabilities at each imputed marker for each individual. In all analyses, only those SNPs with an imputation information/accuracy of r^2^ > 0.30 and a minor allele frequency (MAF) > 0.3 were considered. The haplotypes and their posterior probabilities were estimated using the expectation-maximization algorithm [21]. Only the four *HMMR* haplotypes with the highest probabilities were considered; the rest were grouped into a single rare haplotype. Each carrier was assigned the most likely haplotypes and the association between haplotypes and age at breast cancer diagnosis was evaluated using a standard Cox proportional hazards model. All possible pairwise gene interactions including rs299290 or rs2426618 were evaluated using a standard Cox proportional hazards model that considered the main effects and the interaction term.

### Expression Analysis

The association between gene expression and survival after breast cancer diagnosis was assessed using the NKI-295 dataset of sporadic primary breast tumors [22,23] and a standard Cox proportional hazards model. All possible pairwise gene interactions including *HMMR* or *AURKA* microarray probes (2 and 1 probes, respectively) were evaluated using this model. The quantitative analysis of *HMMR* expression isoforms was carried out using mRNA extracted from lymphoblastoid cell lines of nine rs299290-TT and six rs299290-CC *BRCA2* mutation carriers, and the following TaqMan (Applied Biosystems) probes in real-time PCR assays: hs01063269 for total *HMMR* expression; hs0106328 for the inclusion of exon 4; and hs00234864 for the inclusion of exon 11.

### Genome Analyses

Data for formaldehyde-assisted isolation of regulatory elements (FAIREs) that marked transcriptionally active regions in normal human mammary epithelial cells (HMECs) were downloaded from the Gene Expression Omnibus (GEO) reference GSE46074 [24]. Sequence reads were trimmed for the adaptor, masked for low-complexity and low-quality sequences/reads and subsequently aligned to the genome version hg19 using TopHat [25] with default parameters. Peaks were called using HOMER [26], applying a triangle-based distribution, a median length of 150 base pairs, and an α value of 0.01 (99.0% CI). Replicates were analyzed individually and uniquely merged using BEDTools [27]. Chromatin immunoprecipitation data of ERα were downloaded from the GEO reference GSE32222 and analyzed with MACS (version 2.0.9; macs2diff function) [28]. Significance was defined as a false discovery rate < 1%, using default values for all other parameters. Differentially bound genomic regions were assigned to the closest ENSEMBL (version 62) annotated gene using the R-Bioconductor package ChIPpeakAnno [29]. Histone modification and chromatin segmentation data in HMECs were obtained from the UCSC Genome Browser (hg19) and correspond to the GEO references GSE29611 and GSE38163, respectively, deposited by the ENCODE project [30].

### Evolutionary analysis of BRCA1 and RHAMM

The full-length nucleotide and protein sequences from 20 (for *BRCA1*/BRCA1) and 26 (for *HMMR*/RHAMM) mammalian species, which included human and naked mole rat, were downloaded from the OrthoMaM 2.0 database [31] (**S2 Table**). For evolutionary analysis, a multiple sequence alignment (MSA) of the corresponding amino acid sequences was generated using the algorithm implemented in PRANK v.140110 [32]. To prevent the inclusion of incorrectly aligned positions, all MSA positions with low statistical support (posterior probabilities < 0.99) in the PRANK alignment were excluded. Next, the high-quality protein MSAs were used as guide in the alignment of the corresponding coding sequences (CDS MSAs). The level of functional constraints acting on the coding regions of both genes was analyzed using the maximum likelihood method implemented in the *codeml* program of PAML v.4 [33]; this approach allows to estimate the non-synonymous (*d*_N_) to synonymous (*d*_S_) ratio (ω) in a particular coding region by using a codon-based evolutionary model under a phylogenetic framework, allowing comparison of their fit to the data by the likelihood ratio test (LRT). In particular, the goodness-of-fit of two nested evolutionary models was compared: the M7 model, which assumes a *β* distribution of ω across sites between 0 and 1 (0 ≤ ω ≤ 1); and the M8 model, which adds to M7 an extra category of positively selected sites (ω > 1). To reduce the probability of false positive results from the M7-M8 comparison, we also estimated the likelihood of the data under the model M8a [34], in which ω was set to 1. The posterior probabilities for each site of belonging to the positively selected class were computed using the Bayes empirical Bayes approach in *codeml* [35]. In all models, the topology of the mammalian phylogenetic tree assumed in OrthoMaM database was used (**S2 Table**).

## Results

### *HMMR* rs299290 Association

**Fig 1:**
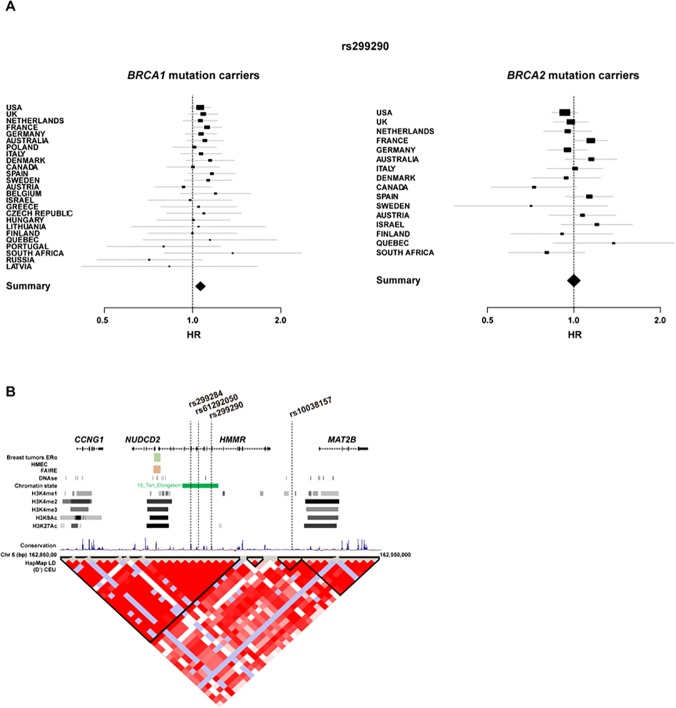
The *HMMR* locus and breast cancer risk in *BRCA1* mutation carriers. (**A**) Forest plots showing rs299290 HRs and 95% CIs (retrospective likelihood trend estimation) for participating countries (relatively small sample sets are not shown) ordered by sample size. Left and right panels show results for *BRCA1* and *BRCA2* mutation carriers, respectively. The sizes of the rectangles are proportional to the corresponding country/study precision. (**B**) The rs299290-containing region, including the genes, variation and regulatory evidence mentioned in HMECs. Exons are marked by black-filled rectangles and the direction of transcription is marked by arrows in the genomic structure. The chromosome 5 positions (base pairs (bp)) and linkage disequilibrium structure from Caucasian HapMap individuals are also shown.

The product of the *HMMR* gene, RHAMM, interacts with BRCA1 in the control of mammary epithelial polarization and this function may be at the basis of a modification of breast cancer risk in *BRCA1* mutation carriers [4]. In this iCOGS *BRCA1/2* study, 14 SNPs (11 with pairwise r^2^ < 0.85) at the *HMMR* locus were genotyped in 15,252 *BRCA1* and 8,211 *BRCA2* mutation carriers from 60 participating centers. Among these variants, the strongest evidence of association with breast cancer risk in *BRCA1* mutation carriers was observed for the originally reported SNP rs299290 [4] (MAF = 0.25): *BRCA1* per-allele HR = 1.10, 95% CI 1.04–1.15, p = 1.9 x 10^−4^ (FDR-adjusted p = 0.043, accounting for 41 genotyped SNPs used in the association analyses in *BRCA1* and *BRCA2* mutation carriers). In contrast, no evidence of association was obtained between *HMMR* variation and breast cancer risk in *BRCA2* mutation carriers; specifically, rs299290 per-allele HR = 0.98, 95% CI 0.92–1.05, p = 0.57. The effect among *BRCA1* mutation carriers was consistent across most participating countries (**Fig. 1A**) and no heterogeneity was detected (p_heterogeneity_ ≥ 0.30). Importantly, the *BRCA1* association remained after excluding the centers participating in the original study [4]: n = 5,039, rs299290 per-allele HR = 1.13, 95% CI 1.04–1.22, p = 0.005.

Although the original Ashkenazi Jewish population study suggested associations involving SNPs proximal to *HMMR* (∼450 kb proximal) [1], no evidence of association in *BRCA1/2* mutation carriers was obtained for five correlated variants in this region (**S1 Table**). With respect to rs299290 and ovarian cancer risk, no evidence of association was found under the single disease risk model or the competing risks model (p > 0.65; only breast cancer risk in *BRCA1* mutation carriers was significant in this model: p = 2.5 x 10^−4^). Together, these results corroborate the association between variation at the *HMMR* locus and breast cancer risk in *BRCA1* mutation carriers.

### Mapping the *HMMR* locus association

Allelic imputation within ∼60 kb centered on *HMMR* (fully including the proximal genes *CCNG1* and *NUDCD2*) did not detect substantially stronger associations than those identified for rs299290: a variant located in *HMMR* intron 7 (rs61292050; **Fig. 1B**) was found to be similarly associated (p = 2.7 x 10^−4^), but this was correlated with rs299290 (r^2^ = 0.95). Haplotype analyses were then carried out to demarcate the *HMMR* genomic region potentially harboring a causative variant or mutation. Using the 14 SNPs genotyped in iCOGS, two haplotypes, both characterized by the minor allele of rs299290, were found to be associated with breast cancer risk in *BRCA1* mutation carriers (**S3 Table**). Based on these haplotypes, the minimal region harboring a mutation could be delimited to ∼28 kb between rs299284 and rs10038157 (**S3 Table**).

### Analysis of the potential causative variant

The rs299290 variant represents a missense amino-acid change in *HMMR* exon 11 that is predicted to be benign/neutral/tolerated by several algorithms: Valine 369 to Alanine in accession number NP_001136028.1; MutationAssessor score = 0; Polyphen score = 0.005; and SIFT score = 0.73. Subsequent examination of the splicing of exon 11 and of exon 4, the latter of which is known to be differentially spliced in different conditions and cell types [36], did not reveal alterations or differences between mRNA samples with different rs299290 genotypes (**S1 Fig.**). Nonetheless, rs299290 is located ∼14 kb from the *HMMR* promoter region that is active in mammary epithelial cells, as detected by the analysis of data from genome occupancy profiling [24] (**Fig. 1B**). In addition, analysis of data for ERα binding plasticity [37] revealed significant binding of this factor at the *HMMR* promoter in poor-prognosis breast tumors (**Fig. 1B**).

**Fig 2:**
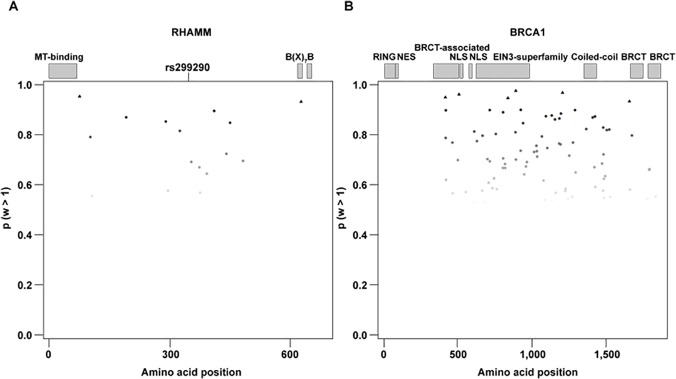
Candidate amino acid sites under positive selection in RHAMM and BRCA1. (**A**) Plot showing the position of potentially selected sites (p (w > 1)) in the amino acid sequence of RHAMM. The relative position of known protein domains is shown. (**B**) Plot showing the position of potentially selected sites (p (w > 1)) in the amino acid sequence of BRCA1. The relative position of known protein domains is shown.

Causal alleles for different common diseases have shown evidence of positive selection [38]. Notably, a recent report suggested the action of positive selection on the evolution of BRCA1 and RHAMM orthologs in the naked mole rat, which is an exceptionally cancer-resistant species [39]. Following on from this suggestion, we identified footprints of positive selection in the evolution of some amino acids of both proteins. In both cases, model M8 (selection model) better fits the protein alignment data than model M7 (null model): p values = 6.69 x 10^−14^ and 1.55 x 10^−5^, for BRCA1 and RHAMM, respectively. Moreover, the likelihood of the data is significantly higher under model M8 than under the nested model M8a: p values = 6.22 x 10^−10^ and 0.014 for BRCA1 and RHAMM, respectively, confirming the presence of positively selected sites in these alignments. However, Valine 369 RHAMM was not identified in these analyses; the predicted amino acid sites under selection were only linked to rare variants (MAFs < 0.01) (**S4 Table**). Nonetheless, as suggested in the analysis of the naked mole rat sequence, Valine 369 is within a region with a potential excess of selected positions (**Fig. 2A**). Analysis of BRCA1 also showed multiple potential selection sites (**Fig. 2B**), but the specific region or domain mediating the interaction with RHAMM remains unknown [1,4,40].

### Evaluation of *HMMR* association by ERα tumor status and *BRCA1* mutation class

The original study suggested an association between the rs299290 risk allele and ERα-negative breast cancer for *BRCA1* mutation carriers [4]. In the present study, no difference was found in the rs299290 effect between ERα-negative and ERα-positive cases: ERα-negative, per-allele HR = 1.09, 95% CI 1.03–1.15, p = 2.3 x 10^−3^; ERα-positive, per-allele HR = 1.09, 95% CI 0.98–1.21, p = 0.13; p_difference_ = 0.96. Interestingly, there was a suggestion of an rs299290 association with ERα-negative breast cancer for *BRCA2* mutation carriers, but in the opposite direction to that observed for *BRCA1* mutation carriers: ERα-negative *BRCA2* mutation carriers n = 434, per-allele HR = 0.83, 95% CI 0.70–0.97, p = 0.022. In addition, there was no evidence of an rs299290 association with ERα-positive breast cancer in *BRCA2* mutation carriers (n = 1,490, p = 0.40, ERα-negative effect p_difference_ = 0.019).

Regarding *BRCA1* mutation classes, the original study [4] suggested a rs299290 association in carriers of mutations expected to result in a reduced transcript or protein level due to nonsense-mediated RNA decay (Class 1), but not in carriers of mutations likely to generate stable proteins with a potential residual or dominant-negative function (Class 2). The current study indicated a similar association, although the estimations were not significantly different: Class 1, rs299290 per-allele HR = 1.11, 95% CI 1.05–1.18, p = 4.6 x 10^−4^; Class 2, rs299290 per-allele HR = 1.03, 95% CI 0.94–1.14, p = 0.51. Regarding Ashkenazi Jewish *BRCA1* mutation carriers (n = 1,231), there were no significant associations in this population or with founder mutations (185delAG HR = 0.94, p = 0.17; and 5382insC HR = 0.85, p = 0.10). Larger sample series may be required to assess associations in these settings and their consistency with previous observations in the Ashkenazi Jewish population [1].

### *AURKA/CSTF1* association with breast cancer risk in *BRCA2* mutation carriers

**Fig 3:**
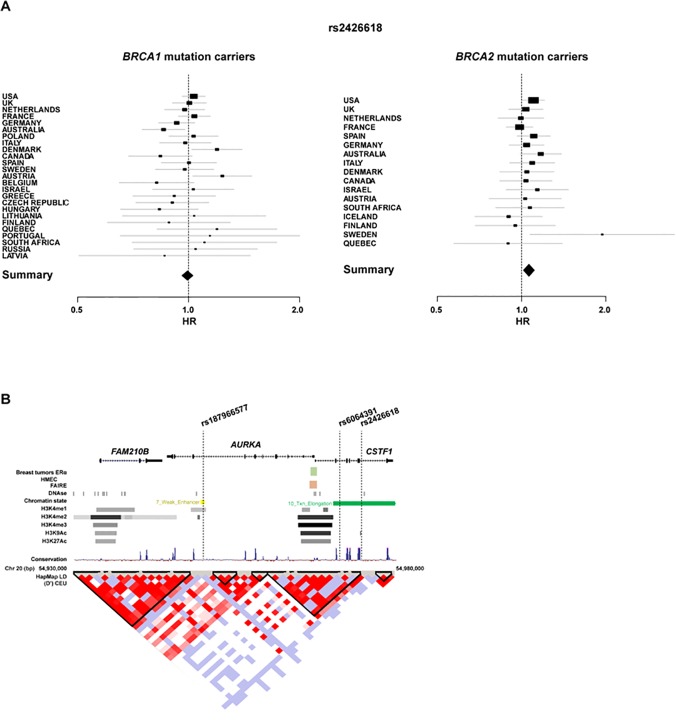
The *AURKA/CSTF1* locus and breast cancer risk in *BRCA2* mutation carriers. (**A**) Forest plots showing rs2426618 HRs and 95% CIs (retrospective likelihood trend estimation) for participating countries (relatively small sample sets are not shown) ordered by sample size. Left and right panels show results for *BRCA1* and *BRCA2* mutation carriers, respectively. The sizes of the rectangles are proportional to the corresponding study precision. (**B**) The rs2426618-containing region, including the genes, variation and regulatory evidence in HMECs. Exons are marked by black-filled rectangles and the direction of transcription is marked by arrows in the genomic structure. The chromosome 20 positions (bp) and linkage disequilibrium structure from Caucasian HapMap individuals are also shown.

As the *HMMR* and *AURKA-TPX2-TUBG1* gene products are functionally related in the regulation of mammary epithelial polarization [4], the associations between variants at these loci that were included on the iCOGS array and cancer risk in *BRCA1/2* mutation carriers were assessed (**S1 Table**). No associations were observed for *TPX2* and *TUBG1*, but there was an indication of an association for a variant relatively close to *AURKA*; rs2426618 and breast cancer risk in *BRCA2* mutation carriers, per-allele HR = 1.10, 95% CI 1.03–1.16, p = 0.005 (FDR-adjusted p = 0.045; **Fig. 3A**). There was no evidence of association between this variant and breast cancer risk in *BRCA1* mutation carriers or between the same variant and ovarian cancer risk in either *BRCA1* or *BRCA2* mutation carriers (p > 0.30). Consistent with the main analysis, the competing risk model showed an rs2426618 association with breast cancer risk only in *BRCA2* mutation carriers (p = 0.002).

The rs2426618 variant is located in intron 5 of a neighboring (distal) gene, *CSTF1* (**Fig. 3B**). Analogous to the *HMMR* setting, rs2426618 is relatively close (∼30 kb) to the promoter region of *AURKA*/*CSTF1*, which is active in mammary epithelial cells and differentially ERα-regulated in poor-prognosis breast tumors (**Fig. 3B**). Association analysis with imputed SNPs within ∼50 kb centered on rs2426618 (fully including *AURKA* and *CSTF1*) did not reveal stronger evidence than that of rs2426618: a variant located in *AURKA* intron 8 (rs187966577, **Fig. 3B**) was found to be similarly associated (p = 0.002). However, this variant was rare (MAF = 0.009) and poorly imputed (r^2^ = 0.37). Finally, the analysis of rs2426618 by ERα tumor status in *BRCA2* mutation carriers did not reveal specific associations: ERα-positive, per-allele HR = 1.10, 95% CI 1.02–1.17, p = 0.010; ERα-negative, per-allele HR = 1.11,95% CI 0.97–1.26, p = 0.15, p_difference_ = 0.89.

### Potential genetic interactions in the *AURKA-HMMR-TPX2-TUBG1* module

Given the above associations at *HMMR* and *AURKA*/*CSTF1*, the influence of GxG on breast cancer risk in *BRCA1/2* mutation carriers was assessed between rs299290 and the genotyped variants in *AURKA/TPX2/TUBG1* (n = 22), and between rs2426618 and the genotyped variants in *TPX2/TUBG1* (n = 7). No GxG was detected for rs2426618-*TPX2/TUBG1*, but potential interactions (unadjusted p_interaction_ values < 0.05) between rs299290 and *AURKA*/*CSTF1* or *TUBG1* variants were suggested (**Table 1**). An interaction between rs299290 and rs6064391 (in intron 2 of *CSTF1*, **Fig. 3B**) could reduce breast cancer risk in both *BRCA1* and *BRCA2* mutation carriers (interaction HRs = 0.87 and 0.73, respectively; **Table 1**). In the main effect analysis, rs6064391 showed a suggestion of association with reduced breast cancer risk in *BRCA2* mutation carriers: HR = 0.87, unadjusted p = 0.036. Conversely, an interaction between rs299290 and rs11649877 (3’ region of *TUBG1*) could increase breast cancer risk in both *BRCA1* and *BRCA2* mutation carriers (HRs = 1.33 and 1.21, respectively; **Table 1**). In the main effect analysis, there was no evidence of rs11649877 association with breast cancer risk in *BRCA1* or *BRCA2* mutation carriers (p = 0.49). Genetic interactions frequently represent complex molecular relationships and, thus, can be explained by multiple models of phenotypic differences across genetic backgrounds [41], including those identified here. However, additional studies are required to corroborate the findings.

**Table 1:** Potential GxG associated with breast cancer risk in *BRCA1/2* mutation carriers.

Genotype(s) (gene locus)	HR*	p_interaction_
***BRCA1* mutation carriers**		
rs299290-A/G (*HMMR*)	1.16	
rs6064391-A/A (*CSTF1*)	1.09	
rs299290-A/G—rs6064391-A/A	0.87	0.047
rs299290-A/G (*HMMR*)	1.07	
rs11649877-A/G (*TUBG1*)	0.94	
rs299290-A/G—rs11649877-A/G	1.33	0.014
***BRCA2* mutation carriers**		
rs299290-G/G (*HMMR*)	1.32	
rs6064391-A/C (*CSTF1*)	0.97	
rs299290-A/G—rs6064391-A/C	0.73	0.034
rs299290-A/G (*HMMR*)	0.95	
rs11649877-A/A (*TUBG1*)	0.87	
rs299290-A/G—rs11649877-A/A	1.21	0.041

*Each estimate is derived from the interaction term of a Cox regression model.

**Fig 4:**
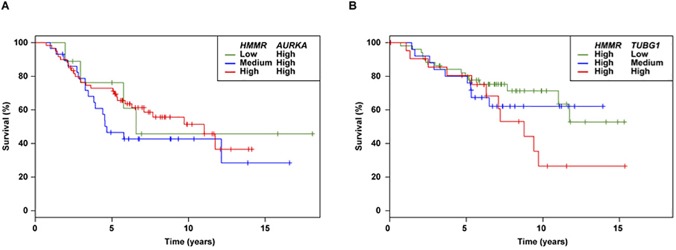
Gene expression interactions in breast cancer survival. (**A**) Kaplan–Meier survival curves based on categorization of *HMMR* (probe NM_012484) and *AURKA* (NM_003600) expression in tertiles (low, medium or high expression). For simplicity, only the tertiles for “high” *AURKA* are shown. The tumours with high expression levels for both genes were not those with the poorest prognosis. (**B**) Kaplan–Meier survival curves based on categorization of *HMMR* (NM_012484) and *TUBG1* (NM_016437) expression in tertiles (low, medium or high expression). For simplicity, only the tertiles for “high” *HMMR* are shown. The cases with high expression levels for both genes were those with the poorest prognosis.

Integrative studies in model organisms have shown that experimentally identified GxG overlap with other types of gene and/or protein interactions (*e*.*g*., gene co-expression) to a degree that is significantly higher than expected by chance [8]. Thus, we assessed the GxGs suggested above for their equivalence with interactions between gene expression profiles across breast tumors, considering breast cancer survival as the outcome. There are no large tumor series from *BRCA1/2* mutation carriers that allow gene expression interactions in these mutation backgrounds to be assessed; therefore our analysis was restricted to sporadic cases and aimed to explore complex relationships between *HMMR* and *AURKA* or *TUBG1*. Using the NKI-295 dataset [22], *HMMR* expression was found to be significantly associated with overall survival: two microarray probes gave identical results: HR = 1.78 and p = 0.001. *AURKA* expression was also found to be associated with overall survival (single probe HR = 2.07, p = 3.3 x 10^−9^) but, notably, the combined model with *HMMR* indicated a protective interaction: the HRs for both *HMMR* and *AURKA* probe combinations were 0.57 and 0.55, p_interaction_ values = 0.026, respectively (**Fig. 4A**). The combined *HMMR* and *TUBG1* expressions also suggested an interaction, but in this case “aggravating”: the HRs for both *HMMR* probe combinations with a *TUBG1* probe were 1.84 and 2.16, p_interaction_ = 0.041 and 0.009, respectively (**Fig. 4B**). The expression of *TUBG1* alone was not found to be associated with overall survival (p = 0.12). Together, these results could indicate a functional correspondence for interactions influencing both breast cancer development and progression. However, the precise effects of the corresponding risk alleles remain to be elucidated.

## Discussion

The results of this study expand on the previous suggestion that variation in *HMMR* is specifically associated with breast cancer risk in *BRCA1* mutation carriers [4]. By analyzing a much larger number of carriers—genotyped using the high-quality iCOGS approach [11,12]—we were able to confirm the association of this locus, and to demonstrate that the strongest signal corresponded to rs299290. No specific effect was revealed when analyzing this association by ERα tumor status. Larger carrier series may be required to comprehensively evaluate associations by tumor subtype or, conversely, to establish that variation in *HMMR* interacts with Class I *BRCA1* mutations to give rise to any type of tumor. In this context, mapping of the protein and/or functional domains interacting between BRCA1 and RHAMM would be necessary to decipher the role of rs299290, if any, and of the observed potential positive selection. With regard to the results obtained for *BRCA2* mutation carriers, the opposite direction of the potential association with ERα-negative breast cancer compared with the association observed for *BRCA1* mutation carriers is intriguing. Opposite effects between cancer subtypes have been observed for other modifier loci [16] and, thus, potentially inform on opposing functional roles in biological processes influencing carcinogenesis. In other words, alteration of RHAMM function in mammary epithelial differentiation may have a differential effect on breast cancer risk depending on whether it occurs in a *BRCA1-* or *BRCA2*-mutated background.

The study of gene loci functionally related to *HMMR* suggests an association between variation in *AURKA*/*CSTF1* and risk of breast cancer among *BRCA2* mutation carriers. While there were initial conflicting results for *AURKA* associations with breast cancer risk [5,42], it is interesting to note that a population case-control study [43] identified variants in the *AURKA* promoter region associated with breast cancer risk in the same direction as detected in our analysis. The minor allele of rs6064389 was shown to be protective in the general population [43], and a similar association was observed in the analysis of *BRCA2* mutation carriers: HR = 0.93, p = 0.021; not significant in *BRCA1* mutation carriers, p = 0.25. This variant is partially correlated with rs2426618 (r^2^ = 0.63), and more strongly correlated with rs6064391 (r^2^ = 0.86), which might interact with rs299290.

In addition to the main effects, interaction between *HMMR* and *AURKA*, and between *HMMR* and *TUBG1* could influence breast cancer risk in *BRCA1* and *BRCA2* mutation carriers. The interactions did not remain statistically significant after corrections for multiple testing and, therefore, additional studies are warranted to investigate these findings further. Combined expression of *HMMR* and *AURKA*, and *HMMR* and *TUBG1* in sporadic breast tumors appeared to influence patients’ survival differentially. These results could be analogous to those observed for breast cancer risk, but it remains unknown how the corresponding risk alleles may alter gene expression and/or protein function. In this context, over-expression of *HMMR*, *AURKA* or *TUBG1* impairs mammary epithelial polarization [4] and the rs299290 risk allele could be associated with relatively higher levels of *HMMR* expression [1,4], which would be consistent with the observed interactions. However, other studies on the potential regulatory impact of this variant and/or the link to selective constraints are needed. It is also important to note that the altered gene products may not be those included in the depicted functional module, but rather other products from the corresponding chromosomal regions.

In summary, centered on the *AURKA-HMMR-TPX2-TUBG1* functional module that regulates mammary epithelial polarization, this study confirms previous association results for *HMMR* rs299290 and suggests novel associations (for *AURKA*/*CSTF1* and *HMMR*-rs299290 interactions) with breast cancer risk in *BRCA1* and/or *BRCA2* mutation carriers.

## Supporting Information

S1 Fig Results of the quantification of the expression of *HMMR* exons 4 and 11 according to the rs299290 major and minor genotypes. (TIF)

S1 Table Genotyped variants and breast cancer association results in *BRCA1* and *BRCA2* mutation carriers. (XLSX)

S2 Table Species and genes used in functional constraints analyses. (XLSX)

S3 Table Variation in *HMMR* and haplotype association study for risk of breast cancer in *BRCA1* mutation carriers. (PDF)

S4 Table Evolutionary selection tests and results. (XLS)

S5 Table Details of acknowledgments and funding support. (XLSX)
