# Supplementary figures and images for: Assessing Associations between the AURKA-HMMR-TPX2-TUBG1 Functional Module and Breast Cancer Risk in BRCA1/2 Mutation Carriers
Source: PLoS One. 2015 Apr 1;10(4):e0120020. doi: 10.1371/journal.pone.0120020 (PMC4382299; doi:10.1371/journal.pone.0120020)

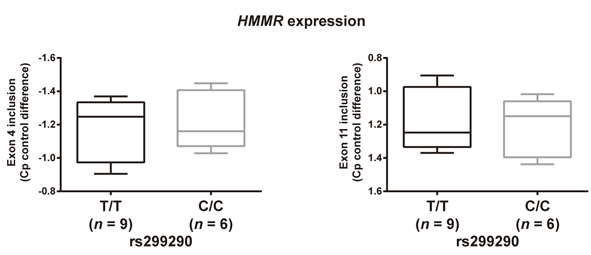

Supplement: S1 Fig — (TIF) [file pone.0120020.s001.tif]
