# Supplementary material for: Assessing Associations between the AURKA-HMMR-TPX2-TUBG1 Functional Module and Breast Cancer Risk in BRCA1/2 Mutation Carriers
Source: PLoS One. 2015 Apr 1;10(4):e0120020. doi: 10.1371/journal.pone.0120020 (PMC4382299; doi:10.1371/journal.pone.0120020)
Supplement: S3 Table — (PDF) [file pone.0120020.s004.pdf]

**Table S3. Variation in *HMMR* and haplotype association study for risk of breast cancer in *BRCA1* mutation carriers (*n* = 14,797)*.**

| Haplotype | Frequency | rs2069368 | rs177250 | rs299302 | rs6877450 | rs2303076 | rs3756648 | rs299284 | rs2303078 | rs299290 | rs299295 | rs299318 | rs299319 | rs10038157 | rs7734424 | HR | 95% CI | *p* |
|---|---|---|---|---|---|---|---|---|---|---|---|---|---|---|---|---|---|---|
| A | 0.46 | G | A | G | A | G | A | G | G | A | G | G | A | A | G | 0.99 | 0.96-1.02 | 0.47 |
| B | 0.22 | G | A | G | A | A | G | G | G | A | G | G | A | A | G | 0.96 | 0.93-0.99 | 0.045 |
| C | 0.11 | G | G | A | A | G | G | A | G | G | A | G | G | A | A | 1.08 | 1.02-1.13 | 0.004 |
| D | 0.10 | G | G | A | A | G | G | G | G | G | A | G | G | G | G | 1.08 | 1.02-1.14 | 0.005 |

*The results are those of a standard Cox proportional hazards model
